# ERK-estrogen receptor α signaling plays a role in the process of bone marrow mesenchymal stem cell-derived exosomes protecting against ovariectomy-induced bone loss

**DOI:** 10.1186/s13018-023-03660-5

**Published:** 2023-03-27

**Authors:** Hui Qi, Enpu Shen, Xiong Shu, Danping Liu, Cheng’ai Wu

**Affiliations:** 1 Beijing Research Institute of Traumatology and Orthopaedics, Beijing, 100035 China; 2 Beijing Jishuitan Hospital, Beijing, 100035 China; 3 Department of Orthopaedics, The First Affiliated Hospital of Jinzhou Medical University, Jinzhou, 121000 China

**Keywords:** Bone marrow mesenchymal stem cell-derived exosomes, Osteoporosis, ERK, Estrogen receptor α

## Abstract

**Background:**

Exosomes derived from bone marrow mesenchymal stem cells (BMSC-Exos) are considered as candidates for osteoporosis (OP) therapy. Estrogen is critical in the maintenance of bone homeostasis. However, the role of estrogen and/or its receptor in BMSC-Exos treatment of OP, as well as its methods of regulation during this process remain unclear.

**Methods:**

BMSCs were cultured and characterized. Ultracentrifugation was performed to collect BMSC-Exos. Transmission electron microscopy, nanoparticle tracking analysis, and western blotting were used to identify BMSC-Exos. We examined the effects of BMSC-Exos on the proliferation, osteogenic differentiation, mineralization, and cell cycle distribution of MG-63 cells. The protein expression of estrogen receptor α (ERα) and the phosphorylation of ERK were investigated through western blotting. We determined the effects of BMSC-Exos on the prevention of bone loss in female rats. The female Sprague–Dawley rats were divided into three groups: the sham group, ovariectomized (OVX) group, and the OVX + BMSC-Exos group. Bilateral ovariectomy was performed in the OVX and OVX + BMSC-Exos groups, while a similar volume of adipose tissue around the ovary was removed in the sham group. The rats in OVX group and OVX + BMSC-Exos group were given PBS or BMSC-Exos after 2 weeks of surgery. Micro-CT scanning and histological staining were used to evaluate the in vivo effects of BMSC-Exos.

**Results:**

BMSC-Exos significantly enhanced the proliferation, alkaline phosphatase activity, and the Alizarin red S staining in MG-63 cells. The results of cell cycle distribution demonstrated that BMSC-Exos increased the proportion of cells in the G2 + S phase and decreased the proportion of cells in the G1 phase. Moreover, PD98059, an inhibitor of ERK, inhibited both the activation of ERK and the expression of ERα, which were promoted by administration of BMSC-Exos. Micro-CT scan showed that in the OVX + BMSC-Exos group, bone mineral density, bone volume/tissue volume fraction, trabecular number were significantly upregulated. Additionally, the microstructure of the trabecular bone was preserved in the OVX + BMSC-Exos group compared to that in the OVX group.

**Conclusion:**

BMSC-Exos showed an osteogenic-promoting effect both in vitro and in vivo, in which ERK-ERα signaling might play an important role.

## Introduction

Osteoporosis (OP) is a common bone disease characterized by decreased bone mass and impaired bone microstructure [1–3]. OP-associated fracture represents a significant burden to both patients’ families and society. Estrogen deficiency is recognized as a risk factor in the pathogenesis of OP. Hormone replacement therapy (HRT) is one of the main methods to prevent postmenopausal OP, but increased risk of breast cancer and endometrial cancer emerge after long-term HRT [4–6].Therefore, novel approaches are still needed for managing postmenopausal OP.

As mesenchymal stem cells (MSCs) have the ability of multi-linage differentiation and are easy to obtain and culture, they have become an important source of stem-cell therapy for OP. Bone marrow mesenchymal stem cells (BMSCs), and adipose-derived mesenchymal stem cells (AdSCs) are the most common sources and are used in more than half of the total studies in this area [7, 8]. Many preclinical investigations have proved that BMSC transplantation in ovariectomized (OVX) model animals (e.g., rats, mice, rabbits, and goats) showed significant improvements in osteogenesis [7, 8]. However, direct application of MSCs is also associated with undesired risks, such as immune responses, disease transmission, or even tumor formation [9]. Therefore, it is important to develop other cell-free approaches, which may avoid these risks but have similar or even better therapeutic activity. Recent research has demonstrated that exosomes derived from MSCs (MSC-Exos) represent an attractive substitute for MSCs [10–12].

Exosomes are nanoscale vesicles ranging from 50 to 150 nm in diameter, which are released from almost all types of eukaryotic cells [13]. Exosomes transmit information between cells, and are widely distributed in many body fluids, such as blood, urine, cerebrospinal liquid, breast milk, and saliva [14]. Exosomes are less immunogenic, as well as being easy to store and deliver [15]. MSC-Exos are also critical in the musculoskeletal system, in which they exert roles in the progression of repair and reconstruction of cartilage and tendon [16, 17]. Moreover, several studies have proven that exosomes accelerate fracture healing in animal models [18, 19]. In prevalent joint diseases, MSC-Exos can be used to treat osteoarthritis (OA) and osteochondral lesions in animal models [20]. We have also found that extracellular vesicles (mostly exosomes) from GPNMB (glycoprotein non-melanoma clone B)-modified BMSCs attenuated bone loss in OVX rats [21]. All of the data demonstrated the therapeutic potential of MSC-Exos for the treatment of bone disease.

The mechanism of MSC-Exos still requires further exploration. In this study, we used rat BMSCs as the source to generate exosomes, verified their effects on the proliferation, osteogenic differentiation, mineralization, and cell cycle distribution of MG-63 cells, and confirmed the role of ERK and estrogen receptor α (ERα) signaling in this process. In OVX rats, we observed that exosomes derived from BMSCs (BMSC-Exos) enhanced bone regeneration and microstructure in OP. Our findings provide a theoretical basis for the biological effects of BMSC-Exos on bone health.

## Materials and methods

### Culturing and identification of BMSCs

As previously described [21–23], BMSCs were flushed from the femurs and tibias of 2-week-old Sprague–Dawley (SD) rats and subjected to density gradient centrifugation to obtain mononuclear cells. The cells were then cultured in α-MEM supplemented with 10% fetal bovine serum (FBS), and 1% penicillin/streptomycin (Gibco, USA) at 37 °C with 5% CO_2_. The medium was changed every 2 days, and the cells were trypsinized and subcultured (1:3 per passage). Cells at the third passage were used for all experiments.

To identify the multi-lineage differentiation potential, as we and other groups reported previously [15, 20, 21, 23], the BMSCs were induced to differentiate by switching to osteogenic, adipogenic, or chondrogenic differentiation medium. Flow cytometry was used to confirm the surface markers of BMSCs [24, 25].

### Extraction and identification of BMSC-Exos

The extraction and identification of exosomes have been described in our previous studies [20, 22]. Briefly, the culture medium of BMSCs was centrifuged at 10,000 g for 20 min. The supernatant was collected, and filtered through a 0.22 μm sterile filter (Millipore, USA). Then, the liquid was centrifuged at 100,000 g for 70 min (HITACHI Limited, Japan), and BMSC-Exos were obtained. The supernatant was centrifuged again at 100,000 g for 70 min to enrich the BMSC-Exos. The BMSC-Exos were then carefully resuspended in phosphate buffered saline (PBS) and the particle size distribution was examined with nanoparticle tracking analysis (NTA) [12]. The morphology of the exosomes was observed with an 80 kV transmission electron microscope (TEM, HT7700, HITACHI, Japan), and exosome-specific proteins CD63, CD81, and TSG101 [12, 26, 27] were detected by western blotting.

### Exosome labeling and uptake assay

According to the instructions, BMSC-Exos were labeled with Dil (Sigma-Aldrich, USA), and the excess Dil dye was removed by supercentrifugation. MG-63 cells and the labeled exosomes were incubated for 4 h, before being fixed with 4% paraformaldehyde for 10 min at room temperature. After fixation, the nuclei were stained with Hoechst 33,342 dye (Beyotime, China), and the cytoskeleton was stained with Phalloidin (Invitrogen, USA) for 15 min at room temperature. The internalization of BMSC-Exos was observed by laser confocal microscopy (Olympus, Japan).

### Cell viability assay

To determine the effect of exosomes on cell viability, MG-63 cells were seeded at a density of 2000 cells/well (three replicates per group) in a 96-well plate and the MEM standard culture medium (containing 10% exosome-depleted FBS) supplemented with different concentrations of exosomes, 20 μg/ml, 40 μg/ml, and 80 μg/ml, or an equal volume of PBS for 24 h, 48 h, or 72 h. Then, 10 μL CCK-8 solution (sigma, USA) was added to each well and the plate was incubated at 37 °C for 2 h. Finally, the absorbance of each well was measured at 450 nm under a microplate reader (Bio-Tek, USA).

### Cell alkaline phosphatase activity and mineralization assay

As described previously [21], MG-63 cells were seeded at a density of 3 × 10^5^ cells/well in a 12-well plate. The role of exosomes was tested by supplementing osteogenic induction medium (OIM; Cyagen Biosciences Inc., China) during cell culture, and additional BMSC-Exos (80 μg/ml) or an equal volume of PBS were added to each well. After 7-day induction, staining for alkaline phosphatase (ALP) was conducted by a BCIP/NBT ALP Color Development Kit (Beyotime, China). After induction for 14 d, the cells were stained with 1% Alizarin red S (ARS) (Solarbio, China). For quantitative mineralization measurement, the stained cells were eluted with 10% cetylpyridinium chloride (Sigma, USA) at room temperature for 20 min and the absorbance value was measured at 570 nm. Images of the cells were captured under a Leica microscope.

### Flow cytometric assay

MG-63 cells were seeded at a density of 2000 cells per well in a 96-well plate. The MEM standard culture medium (containing 10% exosome-depleted FBS) was changed into medium containing 1% FBS, and the cells were cultured for another 24 h. Then, the cells were treated with 80 μg/ml BMSC-Exos or PBS for 12, 24, 48, or 72 h, harvested, resuspended in a propidium iodide–RNase solution, and incubated for 2 h in the dark. Finally, the cells were detected by flow cytometry, and the cell cycle distribution was analyzed using the ModFitLT DNA analysis program (Becton Dickinson, San Jose, CA, USA).

### Western blotting

The procedures for western blotting were described in our previous reports [21, 23]. The following primary antibodies were used for western blotting: ERα (1:500 dilution, Abcam), ERK (1:1000 dilution, Cell Signaling Technology), p-ERK (1:1000 dilution, Cell Signaling Technology), and β-actin (1:5000 dilution, Abcam). The secondary antibody was obtained from Beijing Zhongshan Jinqiao Biotechnology Co., Ltd. (Beijing, China). Briefly, the cells were homogenized at 13,000 rpm for 5 min at 4 °C, and the protein was obtained. The protein (20 μg) was subjected to SDS-PAGE, and electro-transferred to PVDF membrane (Whatman, Maidstone, Kent, UK). The membranes were incubated with different primary antibodies and then secondary antibodies then filmed using a chemiluminescent imaging system (Fusion SL2, Vilber Lourmat, Marne-la-Vallée Cedex, France). The optical density of the protein band was quantified by the ImageJ software (Bethesda, USA). The density of ERα was shown as the relative value to that of β-actin, while the density of P-ERK was shown as the relative value to that of its total kinase, ERK.

### Animal model

All animal care and experiments were approved by the Animal Research Ethics Committee of Beijing Jishuitan Hospital. Eighteen female Sprague–Dawley (SD) rats (10 weeks old, weighing 230–250 g) were purchased from SPF Beijing Biotechnology Co. Ltd. (Beijing, China), and were housed at a 12/12 h light/dark cycle, with a controlled temperature (23–25 °C) and steady humidity (40–60%) in the animal center of Beijing Jishuitan Hospital. Based on our previous study [21], the osteoporosis (OP) model was established by bilateral removal of rat ovaries after adaptive feeding for one week. All rats were randomly divided into three groups (6 rats in each group) as follows: (1) sham group (removal of a similar volume adipose tissue around the ovary); (2) OVX group (bilateral ovaries were removed and treated with 100μL PBS through intravenous injection via the caudal vein at 2 weeks after surgery, once a week for 8 weeks); and (3) OVX + BMSC-Exos group (bilateral ovaries were removed and treated with 100μL BMSC-Exos at the concentration of 1 mg/mL through intravenous injection via the caudal vein at 2 weeks after surgery, once a week for 8 weeks).

### ELISA for bone remodeling markers

The blood samples were obtained by cutting the rat tail at the end of the experiment, transferring the blood samples to a 2 ml tube, and centrifuged at 2600 rpm for 10 min to obtain the serum samples for ELISA. If hemolysis occurred, the serum samples should be eliminated. The concentration of serum samples was evaluated by measuring the levels of Osteocalcin (OCN) and C-terminal telopeptide of type I collagen (CTX-I) according to the procedures of the ELISA kit (CUSABIO, Wuhan, China).

### Microcomputed tomography (micro-CT) and histological analyses

The right femurs were removed and fixed in 4% paraformaldehyde for 72 h. Three femurs were sent for micro-CT analyses. A Skyscan 1172 (Belgium) was used to scan the femurs at a resolution of 9 μm per pixel. The cylindrical region started from a distance of 1 mm extending to 4 mm below the growth plate of the distal femur was analyzed to evaluate the bone mass and microstructure. Bone parameters, including bone mineral density (BMD), bone volume/tissue volume fraction (BV/TV), trabecular thickness (Tb.Th), trabecular number (Tb.N), and trabecular separation (Tb.Sp), were carried out with the software of CT-Analyser Version1.11.

After fixation, all the 6 right femurs in each group (including the 3 femurs for micro-CT) were decalcified in 10% EDTA solution (Sigma) for approximately 1 month, before dehydrating, paraffin embedding, and sectioning into 4 μm slices. Hematoxylin–eosin (H&E) staining was used to show the bone trabecular structure. Immunohistochemistry was performed to determine the ERα expression in vivo among different groups and evaluate the function of exosomes in terms of ERα expression. The sections were dehydrated, the antigens were extracted, blocked, and treated with primary ERα (1:200, Abcam) antibody at 4 °C overnight. Then, DAB solution was added after 30 min of incubation with secondary antibody at room temperature. Finally, the images were observed using an optical microscope.

### Statistical analyses

Quantitative data are presented as the mean ± standard deviation. SPSS (version 21.0, SPSS Inc.; Chicago, Ill) was used to perform all statistical analyses. The Student’s *t*-test was used to compare the differences between the two groups. The differences among three or more groups were examined by one-way analysis of variance (ANOVA), and the subsequent between-group differences were confirmed by Tukey’s test. A *P*-value < 0.05 was considered significant.

## Results

### Characterization of BMSCs and BMSCs-Exos

**Fig. 1:**
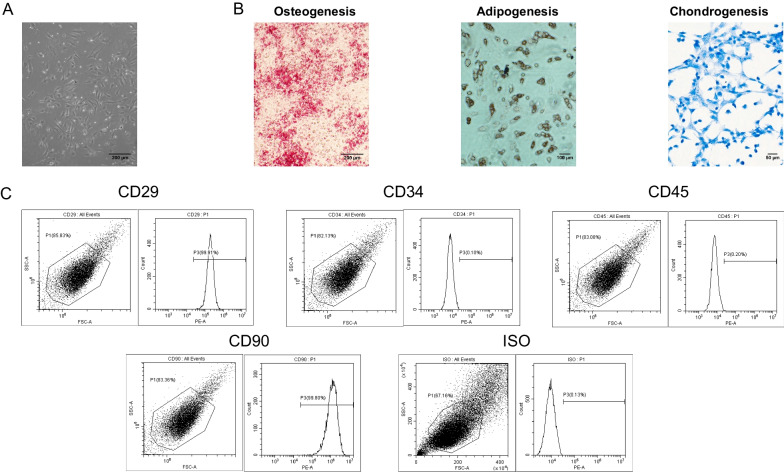
Characterizations of bone marrow stem cells (BMSCs) and exosomes derived from BMSCs. **A** Light microscopy images demonstrating the typical fibroblast-like morphology of BMSCs. Scale bar. 200 μm. **B** Representative images of the tri-lineage differentiation capacity of BMSCs. Scale bars. 200 μm (left); 100 μm (middle); 50 μm (right). **C** Flow cytometric analysis of BMSC surface markers

BMSCs exhibited characteristics of MSCs, including a spindle-like shape (Fig. 1A) and differentiation capacity for osteogenesis, adipogenesis, and chondrogenesis (Fig. 1B). Flow cytometric analysis showed that BMSCs had positive expression of CD29 and CD90, and negative expression of CD34 and CD45 (Fig. 1C).

**Fig. 2:**
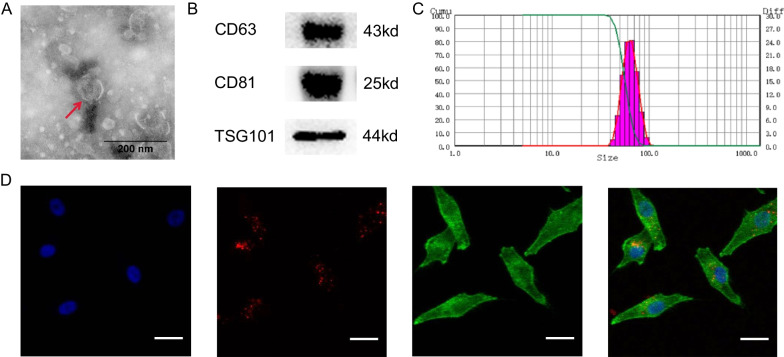
Identification of BMSC-Exos, and the uptake of BMSC-Exos by MG-63. **A** Transmission electron microscope images of the morphology. Scale bar: 200 nm. **B** Detection of CD63, CD81, and TSG101 by western blotting. **C** Nanoparticle tracking analysis. **D** Uptake of BMSC-Exos observed by confocal microscopy. Scale bar: 50 μm

TEM, western blotting, and NTA were used to identify the BMSC-Exos. The hollow spherical microvesicles (Fig. 2A) exhibited exosomal markers, including CD63, CD81, and TSG101 (Fig. 2B). The size distribution of most exosomes ranged from 50 to 120 nm (Fig. 2C), which was consistent with other reports [15, 28]. The data indicated that the microvesicles derived from BMSCs were successfully obtained, and were predominantly exosomes.

### Proliferation, differentiation, and mineralization of MG-63 cells were promoted by BMSCs-Exos

As BMSC-Exos must enter target cells before they can exert their effects, we detected whether BMSC-Exos could be taken up by MG-63 cells. Dil-labeled BMSC-Exos were endocytosed by MG-63 cells, which was observed under a confocal microscope (Fig. 2D).

**Fig. 3:**
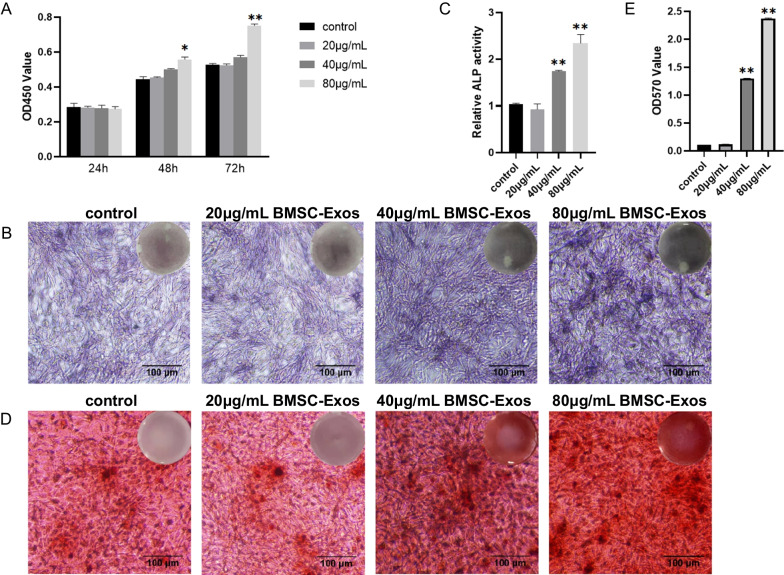
Responses of MG-63 cells co-cultured with BMSC-Exos. **A** Proliferation of MG-63 cells was detected by CCK-8 assay. **B**, **C** Differentiation of MG-63 cells was detected by ALP test kit. Scale bar: 100 μm. D, E. Mineralized nodules in MG-63 cells were detected by Alizarin red S staining. Scale bar: 100 μm. (n = 3 for each group. *P < 0.05 compared to the control. **P < 0.01 compared to the control.)

The CCK-8 assay was used to assess the proliferation of MG-63 cells in vitro. According to the results shown in Fig. 3A, exosomes at a concentration of 80 μg/ml promoted the proliferation of MG-63 cells compared to the control group after co-culturing for 48 h and 72 h (*P* < 0.05 or *P* < 0.01).

ALP activity is an accepted marker of osteoblastic differentiation. As shown in Fig. 3B and C, BMSC-Exos at concentrations of both 40 μg/ml and 80 μg/ml significantly increased the ALP activity of MG-63 cells (*P* < 0.01).

Bone mineralized nodule formation is crucial at the end stage of osteoblastic differentiation [29]. In our experiment, the mineralized nodules were stained with ARS. Similarly, BMSC-Exos also increased the mineralized nodule formation at concentrations of 40 μg/ml and 80 μg/ml (*P* < 0.01) (Fig. 3D and E). The results shown in Fig. 3 clearly indicated that BMSC-Exos exerted the most prominent effects of promoting proliferation, differentiation, and mineralization of MG-63 cells at the concentration of 80 μg/ml; therefore, we used this concentration for the subsequent in vitro experiments.

### Cell cycle distribution of MG-63 cells was altered by BMSC-Exos

**Fig. 4:**
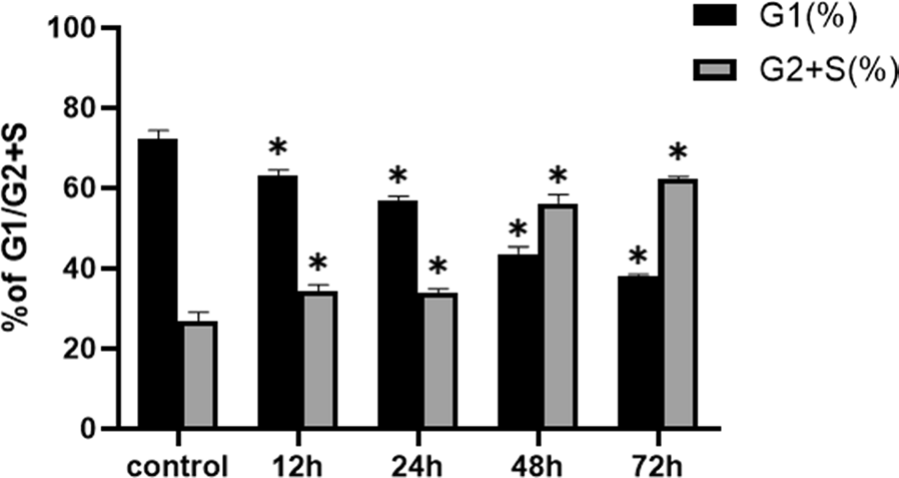
Changes in the cell cycle distribution of MG-63 cells co-cultured with BMSC-Exos. The cells were treated with 80 μg/ml BMSC-Exos for 12 to 72 h. Flow cytometric analysis was used to detect the cell cycle distribution. (n = 3 for each group. *P < 0.05 compared to the control.)

To confirm the effects of BMSC-Exos in vitro, we analyzed the cell cycle distribution of MG-63 cells by flow cytometry. As shown in Fig. 4, 80 μg/ml BMSC-Exos significantly increased the proportion of cells in the G2 + S phase and decreased the proportion of those in the G1 phase. This result indicated that BMSC-Exos might upregulate the proliferation of MG-63 cells by altering the cell cycle distribution.

### BMSC-Exos activated ERK in MG-63 cells

**Fig. 5:**
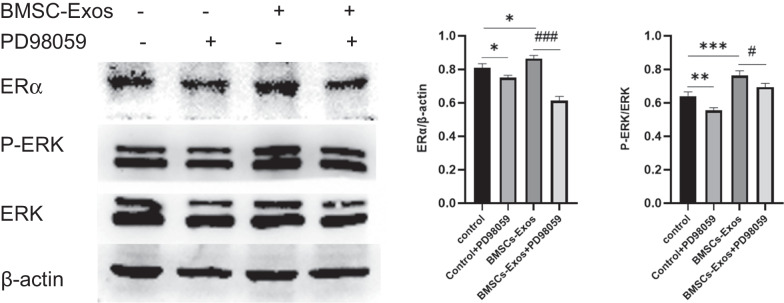
Effects of BMSC-Exos on the protein expression of ERα, P-ERK, and ERK of MG-63 cells. (n = 5 for each group. *P < 0.05, **P < 0.01, ***P < 0.001 compared to the control. ^#^*P* < 0.05 and ^###^*P* < 0.001 compared to BMSC-Exos.)

Evidence has shown that the ERα is an important factor in bone homeostasis [30, 31], and the phosphorylation of ERK can promote osteoblastic differentiation [32–34]. Besides, there is close relationship between ERK and ERα signaling pathway [30, 31, 35, 36]. However, the interaction between ERK and ERα in osteogenesis of BMSC-Exos remains unclear. To elucidate this mechanism, we analyzed the protein expression of ERK and ERα in MG-63 cells. As a result, the expression of ERα was significantly upregulated following the addition of BMSC-Exos (*P* < 0.05) (Fig. 5). A similar result was found by measuring the phosphorylation of ERK (*P* < 0.001). Additionally, the upregulation of ERα and phosphorylation of ERK were both significantly downregulated by PD98059, an inhibitor of ERK. Therefore, we suggested that the ERK-ERα signaling might take part in the effects of BMSC-Exos.

### Bone remodeling markers were altered with BMSC-Exos in OVX rats

**Fig. 6:**
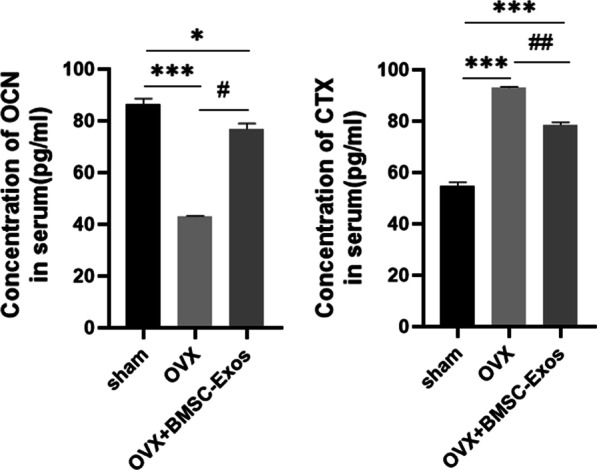
Concentration of OCN and CTX-I in the serum of rats. *P < 0.05 and ***P < 0.001 compared to the sham group. (n = 3 for each group. ^*#*^*P* < 0.05 and ^*##*^*P* < 0.01 compared to the OVX group.)

OCN is a marker of bone formation, while the CTX-I is a marker of bone resorption [37]. The results in Fig. 6 showed that the level of OCN in the OVX group was significantly lower than that of the sham group (*P* < 0.001), but recovered partially after BMSC-Exos treatment (*P* < 0.05); in contrast, the level of CTX-I was significantly higher than that of the sham group (*P* < 0.001), but was promoted following treatment with BMSC-Exos (*P* < 0.01).

### BMSC-Exos eased bone loss in OVX rats

**Fig. 7:**
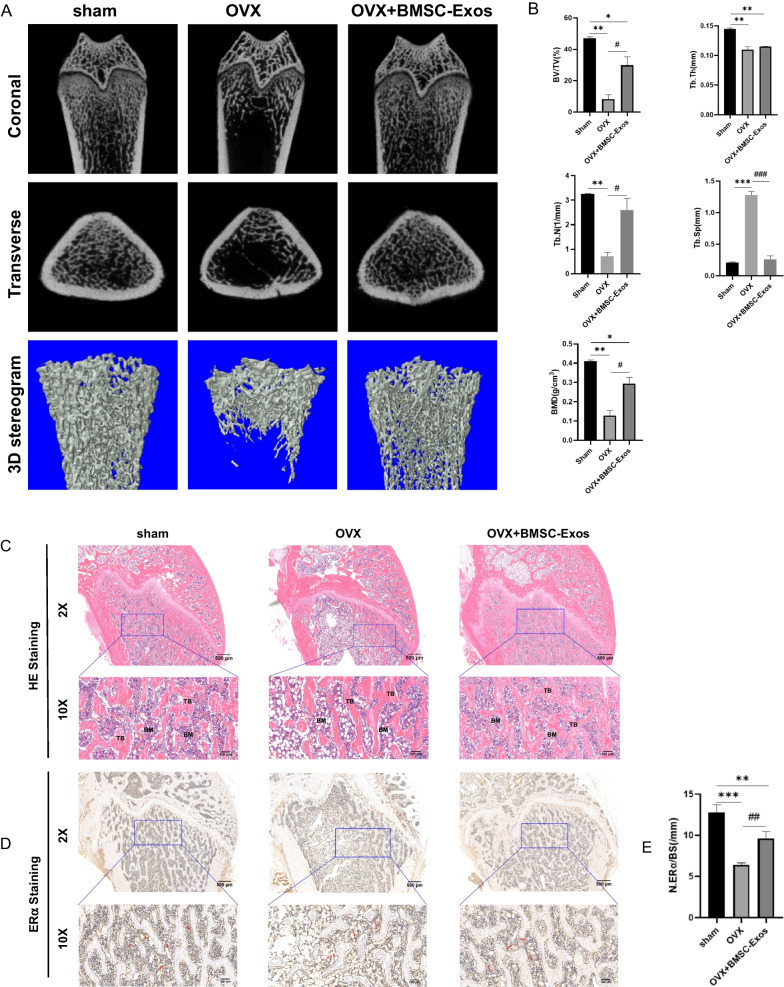
Effects of BMSC-Exos on the OVX-rats. **A** Micro-CT and 3D stereogram of the distal femur in rats. **B** Quantitative analysis of the bone volume/tissue volume fraction (BV/TV), trabecular thickness (Tb. Th), trabecular number (Tb. N), trabecular separation (Tb. Sp), and bone mineral density (BMD). (n = 3 for each group. *P < 0.05, **P < 0.01, and ***P < 0.001 compared to the sham group. ^*#*^*P* < 0.05 and ^*##*^*P* < 0.001 compared to the OVX group.) **C** HE staining images of the femur in rats (TB: Trabecular bone, BM: Bone marrow). **D** ERα immunohistochemical staining images of the femur in rats. **E** Quantitative analysis of the number of ER-positive cells/bone surface (N.ERα/BS). (n = 6 for each group. **P < 0.01 and ***P < 0.001 compared to the sham group. ^*##*^, *P* < 0.001 compared to the OVX group.)

To further determine the effect of BMSC-Exos on bone loss in vivo, we established the rat OVX model and assessed the bone microarchitecture by micro-CT scanning. As a result, the morphology of the femur was significantly improved after treatment with exosomes compared to that observed in the OVX group, and was comparable with the sham group (Fig. 7A). Assessment of the cancellous bone architecture demonstrated that the OVX group had the lowest BMD, BV/TV (%), and Tb.N (1/mm), and the data were significantly increased in the OVX + BMSC-Exos group (*P* < 0.05). In contrast, the Tb.Sp (mm) was the highest in the OVX group, and was significantly decreased following administration of BMSC-Exos (*P* < 0.001) (Fig. 7B). Thus, treating OVX rats with BMSC-Exos could help to reduce the alterations in bone microarchitecture.

Consistent results were found with H&E staining. Compared to the OVX group, a markedly enhanced trabecular structure was shown in the OVX + BMSC-Exos group (Fig. 7C). The immunohistological staining of ERα indicated that the decreasing ERα expression in OVX rats partially recovered following treatment with BMSC-Exos (*P* < 0.01) (Fig. 7D and E), confirming the role of ERα in the effect of BMSC-Exos.

## Discussion

Previous work has suggested that stem cell-derived exosomes are beneficial in the osteoporotic process [14, 38, 39]. Our recent study also demonstrated better bone protection results following the administration of extracellular vesicles derived from GPNMB-modified BMSCs (mostly exosomes) in OVX rats [21]. In this research, we used MG-63 cells in vitro and OVX rats in vivo to examine the osteogenic effects of BMSC-Exos and explored the possible underlying mechanisms. MG-63 cells have been well characterized as an evaluation model for osteoblastic functions [40]. Our findings demonstrated that BMSC-Exos significantly promoted the proliferation, differentiation, mineralization and the proportion in the G2 + S phase of MG-63 cells in vitro, and improved the regeneration of osteoporotic trabecular bone in vivo in OVX rats.

OP is a common skeletal disease, primarily affecting the elderly population, with approximately 200 million people globally estimated to have been impacted by OP [1–3]. The incidence of OP commonly occurs in postmenopausal women due to estrogen deficiency [41, 42]. Thus, the estrogen receptor is a target for the prevention and treatment of bone loss in postmenopausal women [43]. As a result, HRT and selective estrogen receptor modulators (SERMs) have been used clinically; however, these can be associated with severe side effects, including thromboembolic disorders, vasomotor symptoms, and an increased risk of uterine cancer [44]. Considering the safety and effect, novel strategies should be developed for treating OP, with a growing body of evidence suggesting that MSCs may be a promising choice [7, 8].

The paracrine effects of MSCs, especially MSC-Exos, are important in the therapeutic activity of MSCs [14, 38, 39]. In this study, an OVX animal model was used as an OP model, in which the changes after ovariectomy, such as low BMD, microstructural degradation of cancellous bone, and abnormal bone turnover status of serum biomarkers, are all similar to those of osteoporotic patients. Our results of the micro-CT showed that after treatment with BMSC-Exos, the quantitative data, including BMD, BV/TV, Tb.N, and Tb.Sp, were eased significantly. Additionally, the H&E staining images were consistent with the micro-CT data, in which the OVX group experienced remarkable trabecular loss, while BMSC-Exos treatment exhibited preservation of the trabecular bone, and the microstructure of cancellous bone was better in the exosome-treated group than that in the OP group. The serum biomarker determination also demonstrated that BMSC-Exos resulted in effective bone turnover status in OVX rats. Taken together, these data suggest that BMSC-Exos preserved bone formation under osteoporotic conditions.

The osteogenic function of BMSC-Exos was also evaluated in vitro. As a result, we found that the viability of MG-63 cells was obviously increased after the application BMSC-Exos. Meanwhile, BMSC-Exos not only upregulated ALP activity, but increased ARS staining, both of which are characteristic of osteoblasts [45, 46]. Encouragingly, the cell proliferation was also promoted by BMSC-Exos. Since challenges remain in the application of MSCs clinically, namely the likelihood of tumor formation [9], both the in vivo and in vitro effects of BMSC-Exos propose the possibility of BMSC-Exos as a promising substitute for BMSCs in treating OP.

Despite the strengths of this study, the exact mechanisms of BMSC-Exos require further exploration, including whether estrogen and its receptor are involved. Estrogen has fundamental pro-skeletal properties [47], which may inhibit the apoptosis of osteoblasts and simultaneously increase osteoclast apoptosis to promote bone formation [3, 48]. In this study, compared to the control group, the protein expression of ERα increased significantly after co-culturing MG-63 cells with BMSC-Exos. Furthermore, according to the immunohistological results, the positive staining of ERα was promoted in the OVX rats with the treatment of BMSC-Exos. These results highlight the importance and association of ERα in the function of BMSC-Exos. Previous reports have shown that the ERK signaling pathway is involved in the regulation of the biological behavior of osteoblasts and osteoclasts, such as proliferation and apoptosis [32–34]. We also proved that ERK played an important role in the molecular signaling of BMSC-Exos in chondrocyte apoptosis [23]. In this study, PD98059 (an inhibitor of MEKK) could significantly inhibit the activation of ERK. At the same time, the high expression of ERα after treated with BMSC-Exos was also downregulated by PD98059, which demonstrated that ERK-ERα signaling might play a role in the effect of BMSC-Exos. Moreover, we detected the cell cycle of MG-63 cells and found an increase in the G2 + S phase and a decrease in the G1 phase in a time-dependent manner with BMSC-Exos. Therefore, the regulation of the cell cycle may also be involved in the application of BMSC-Exos in the treatment of osteogenesis, but further work is still warranted.

Although the significant bone preservation of BMSC-Exos was indicated in this study, the effect of bone formation was still limited in OVX rats. The exact reason for this is unclear. We speculate that it is much more complicated for an in vivo study, with several factors to be considered, including the specific time point of ending the osteoporotic experiment and the treatment duration, and the dosage and the time of applying exosomes, all of which will influence the results of the in vivo study. Additionally, it is possible that animal species and age are disturbing factors, all of which require further investigation in the future.

## Conclusion

In summary, BMSC-Exos helped to promote the proliferation, DNA synthesis, ALP activity, and mineralization in MG-63 cells. Moreover, BMSC-Exos inhibited the bone loss and preserved the bone microstructure in OVX rats. Although the ERK- ERα signaling pathway may play a role in this process, other pathways should not be neglected.

## Data Availability

We state that the data will not be shared since all the raw data are present in the figures included in the article.
